# Comparative Analysis of Flavonoid Metabolites in Foxtail Millet (*Setaria italica*) with Different Eating Quality

**DOI:** 10.3390/life11060578

**Published:** 2021-06-18

**Authors:** Yakun Zhang, Jianhua Gao, Qianru Qie, Yulu Yang, Siyu Hou, Xingchun Wang, Xukai Li, Yuanhuai Han

**Affiliations:** 1 College of Agriculture, Shanxi Agricultural University, Jinzhong 030801, China; zyakun0809@163.com (Y.Z.); 17835422926@163.com (Q.Q.); yangyulu@stu.sxau.edu.cn (Y.Y.); bragren123@163.com (S.H.); 2 College of Life Sciences, Shanxi Agricultural University, Jinzhong 030801, China; gaojh_edu@163.com (J.G.); wxingchun@163.com (X.W.)

**Keywords:** foxtail millet, flavonoid metabolome, functional components, UPLC-ESI-MS/MS

## Abstract

Foxtail millet (*Setaria italica*) is an important minor cereal crop in China. The yellow color of the de-husked grain is the most direct aspect for evaluating the foxtail millet quality. The yellow pigment mainly includes carotenoids (lutein and zeaxanthin) and flavonoids. To reveal the diversity and specificity of flavonoids in foxtail millet, we chose three high eating quality and two poor eating quality varieties as research materials. A total of 116 flavonoid metabolites were identified based on Ultra Performance Liquid Chromatography-Electrospray Ionization-Tandem Mass Spectrometry (UPLC-ESI-MS/MS) system. The tested varieties contained similar levels of flavonoid metabolites, but with each variety accumulating its unique flavonoid metabolites. A total of 33 flavonoid metabolites were identified as significantly discrepant between high eating quality and poor eating quality varieties, which were mainly in the flavonoid biosynthesis pathway and one of its branches, the flavone and flavonol biosynthesis pathway. These results showed the diversified components of flavonoids accumulated in foxtail millets and laid the foundation for further research on flavonoids and the breeding for high-quality foxtail millet varieties.

## 1. Introduction

Foxtail millet (*Setaria italica* (L.) P. Beauv), a member of the family of *Poaceae*, originated in the Yellow River basin in China more than 11,000 years ago [1]. Foxtail millet plays a nutritional and functional role in the diet for many people [2]. It is nutritionally comparable to some other major cereals. For example, it has a higher content of protein, lipids, and lower carbohydrate content than some cereals (wheat and maize) [3]. Compared with rice, foxtail millet has double protein content, fourfold minerals and fat, and triple calcium [4]. Ample evidence showed that increasing the consumption of foxtail millet was associated with a lower risk of diabetes [5]. Therefore, foxtail millet is gaining increasing attention among consumers. Generally, the yellow color of the de-husked grain is the most direct indicator for consumers to evaluate the millet quality. Many compounds, including carotenoids (lutein and zeaxanthin), flavonoids, are predominant yellow pigments in plants and are believed to confer nutritional and pharmacological benefits to plants. There have been preliminary studies on carotenoids in foxtail millet [6,7]. However, a systematic understanding of flavonoid diversity in foxtail millet is still lacking.

Foxtail millets are rich in flavonoids [8,9], a large group of natural products with variable phenolic structures, containing flavone, flavanone, flavonols, isoflavone, anthocyanins, and so on. Flavonoids play essential roles in plants in forming the color and flavor of flowers and fruits, and resistance against ultraviolet B (UV-B), diseases, and pests damages [10,11,12]. For example, the accumulation of flavonoids led to the deep yellow coloration of red Chinese pears after methyl jasmonate (MeJA) treatment. It also affected the appearance of radishes with multiple colors [13,14]. Furthermore, flavonoids have potential benefits for human health, including antioxidation, anti-inflammatory, anti-cholinesterase, anticancer and heart diseases, and countering antibiotic resistance [15,16,17]. The flavonoid biosynthesis pathway has been well understood in *Arabidopsis thaliana*, *Zea mays*, and *Petunia hybrida*, providing a basis for studying flavonoids in foxtail millet [18,19].

Metabolomics has been introduced as a novel technology to analyze the microbial, plant, and animal metabolomes and is considered the bridge linking the genomes and the phenotypes [20]. The high sensitivity and fast scanning speed make ultra-performance liquid chromatography-tandem mass spectrometry (UPLC-MS/MS) a preferred method of component analysis, in which the multiple reaction monitoring (MRM) mode in triple quadrupole mass spectrometry (UPLC-QQQ-MS/MS) is often used to eliminate the influence of matrix effects in quantitative analyses [21]. Various metabolites and the corresponding genetic characteristics in plants have been identified based on this metabolic path-based approach, such as the identification of glucosides in *Arabidopsis* leaves [22], the synthesis and regulation of flavonoids in rice [23], and the comparison of metabolism substances in plants [24].

This study aimed to characterize flavonoid metabolites in foxtail millets and explore the diversity of flavonoid metabolites among different varieties using UPLC-ESI-MS/MS. It is expected that our work could provide a basis for further understanding of the quality of foxtail millet and pave the way for the breeding of better varieties with high flavonoids.

## 2. Materials and Methods

### 2.1. Plant Materials

Five foxtail millet varieties were selected as experimental materials, which were “Jingu21” (JG21), “Qinzhouhuang” (QZH), “Yugu1” (YG1), “Daobaqi” (DBQ) and “Niumaobai” (NMB). JG21 is an elite variety from Shanxi province with excellent quality and the largest growing area in China. QZH is another outstanding variety bred from an elite landrace that originated from Qinxian County in Shanxi province. YG1 is an elite variety originating from Henan province in China, the most influential species in North China, and has a high-quality reference genome sequence [25]. Both DBQ and NMB are landrace with poor eating quality. All the foxtail millet varieties were planted in the experimental field of Shanxi Agricultural University (37.42 N, 112.59 E), Taigu, China, in May 2018 and harvested in October 2018 at full maturity. The region has a semiarid climate, with an average annual temperature of 11.91 °C and annual precipitation of 396.24 mm in 2018. 

### 2.2. Photo Recording and Color Determination

The husk of foxtail millet seeds was removed using small hulling separators. For each variety, we selected well-developed grains for the experiments. The de-husked millets were put into containers for taking photos. The colors of these millets were measured by a colorimeter (X-Rite VS450, Big Rapids, Michigan, USA). After determining the color, the freeze-dried seed was crushed using a mixer mill (Retsch MM400, Dusseldorf, North Rhine-Westphalia, Germany) with a zirconia bead for 1.5 min at 30 Hz. The powder was also photographed, and its color was measured. Three replicates were carried out. The values of color parameters “L,” “a,” and “b,” which represent the luminosity, redness, and yellowness, respectively, were collected. The color contribution index (CCI) values were calculated according to CCI = 1000 × a/(L × b) [26].

### 2.3. Preparation and Extraction of Samples for Metabolomics Analysis

The preparation and extraction of samples were performed as previously described [27]. One hundred mg foxtail millet powder was weighed and extracted overnight at 4 °C with 1.0 mL 70% aqueous methanol (including 0.1 mg/L lidocaine or 0.1 mg/L acyclovir) to ensure sufficient reaction. After that, the samples were centrifuged at 4 °C and 14,000 r for 5 min. The supernatant was collected and centrifuged again. Then, the water-soluble extracts were absorbed on a CNWBOND Carbon-GCB SPE Cartridge (ANPEL, Shanghai, China) and filtrated with SCAA-104 (ANPEL, 0.22 μm pore size, Shanghai, China), for standby application.

### 2.4. Ultra-High-Performance Liquid Chromatography (UPLC) Conditions

The extracted samples were analyzed as described by Wang et al. [28]. A UPLC-ESI-MS/MS system (UPLC, Shim-pack UFLC SHIMADZU CBM30A system; MS, Applied Biosystems 6500 Q TRAP, Foster City, USA) equipped with a C18 chromatographic column (Waters ACQUITY UPLC HSS T3, 2.1 mm × 100 mm, 1.8 μm) was used for the analysis. The solvent systems contained mobile phase A (0.04% acetic acid in water) and mobile phase B (0.04% acetic acid in acetonitrile). The gradient program (Mobile phase A: Mobile phase B) was performed as follows: 95:5 (*v*/*v*) at 0 min, 5:95 (*v*/*v*) at 11.0 min, 5:95 (*v*/*v*) at 12.0 min, 95:5 (*v*/*v*) at 12.1 min, and 95:5 (*v*/*v*) at 15.0 min; flow rate 0.40 mL/min. The column temperature was kept at 40 °C, and the injection volume was set to 2 μL. The UPLC effluent was input into an ESI-triple quadrupole-linear ion trap (Q TRAP)-MS and analyzed further.

### 2.5. Electrospray-Triple Quadrupole-Linear Ion Trap Mass Spectrometry (ESI-Q TRAP-MS/MS) System

The mass spectrometry (MS) followed the method described by Chen et al. [29]. The Linear ion trap (LIT) and triple quadrupole (QQQ) scans were performed on a triple quadrupole-linear ion trap mass spectrometer (Q TRAP, AB Sciex, Foster City, CA, USA), API 6500 Q TRAP LC/MS/MS System. This system was equipped with an ESI Turbo Ion-Spray interface, with both positive and negative ion modes controlled by Analyst 1.6. The operating parameter of ESI was an ion source (turbo spray, 550 °C, 5500 V). Ion source gas I (GS I), ion source gas II (GS II), and curtain gas (GRU) were set at 55, 60, and 25 psi, respectively. The collision gas (CAD) was set to high. Polypropylene glycol solutions with concentrations at 10 μmol/L and 100 μmol/L were used for instrument debugging and mass calibration in QQQ and LIT modes, respectively. QQQ scanning was performed in a multiple reaction monitoring (MRM) mode with the collision gas (nitrogen) set to 5 psi. The de-clustering potential (DP) and collision energy (CE) for each MRM transition were completed by further optimizing DP and CE. The specific MRM for each time quantum was detected according to the metabolites eluted in this period.

### 2.6. Qualitative and Quantitative Analysis of Metabolites

The qualitative analysis of the primary and secondary spectral data of the MS was analyzed based on the MWDB database (Metware Biotechnology Co., Ltd. Wuhan, China). It removed the repetitive signals containing K^+^, Na^+^, NH4^+^, and fragment ions with larger molecular weight. The quantitative analysis was performed using MRM analysis of QQQ mass spectrometry. After induced ionization in the collision chamber, the precursor ions were fractured into fragments and then filtered through the triple quadrupole in which the desired characteristic fragments were selected. After obtaining the mass spectrometry data, the chromatographic peaks of all the targets were integrated for quantitative analysis [28,30].

### 2.7. Quality Control Analysis of Samples

The stability of the instruments ensured the repeatability and reliability of the data. The quality control samples were composed of mixing equal parts extractions of three groups of different millet varieties. The method of processing and testing samples was the same as that of analyzing the samples. A quality control sample was inserted between 10 testing samples to guarantee the repeatability of the whole analytic process.

### 2.8. Statistical Analysis

Three biological replicates were carried out for each experiment. The significant difference between the groups was determined using a one-way analysis of variance (ANOVA) and Duncan’s multiple range test (*p* < 0.05). Principal components analysis (PCA), orthogonal partial least squares discrimination analysis (OPLS-DA), hierarchical clustering analysis (HCA), and the volcano plot drawing were carried out using ggplot2, muma, pheatmap packages of R (http://www.r-project.org, accessed on 2019) [31,32,33].

## 3. Results

### 3.1. The Millet Color of Different Varieties

**Figure 1:**
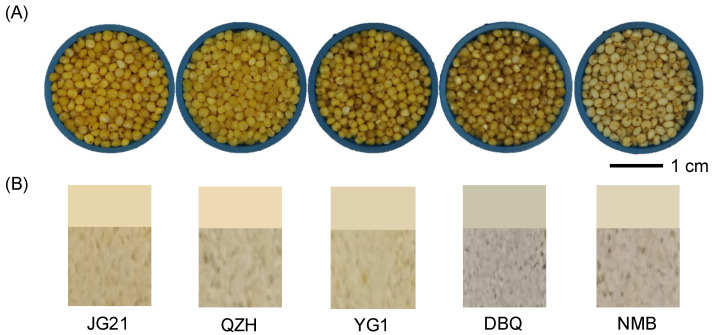
Samples of foxtail millet varieties. (**A**) The foxtail millet kernel samples. (**B**) The foxtail millet powder samples. The top of (**B**) shows the powder of 5 foxtail millet varieties taken by the camera, and the bottom of (**B**) shows the color of foxtail millet powder scanned by the colorimeter.

It is generally believed the darker the yellow color is, the better the eating quality of the millet [34]. To explore the color variation of different quality foxtail millet varieties, we conducted the color measurement of the grains and their matching powder. In the tested varieties, the kernels of JG21, QZH, and YG1 were of different shades of yellow. NMB was a white millet variety, and DBQ was a millet variety with greyish green color (Figure 1A). The powder of JG21 showed the deepest yellow, and DBQ and NMB were both lighter yellow (Figure 1B).

**Figure 2:**
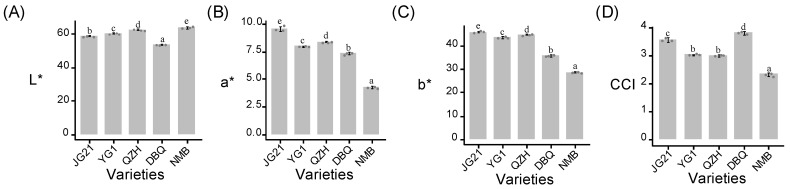
Barplot of color parameters in different foxtail millet varieties. (**A**) Luminosity of millet color. (**B**) Redness of millet color. (**C**) Yellowness of millet color. (**D**) A composite indicator of millet color calculated according to CCI = 1000 × a/(L × b). The x-axis shows the variety name, and the y-axis shows the value of the parameter. The lowercase letters above the histogram indicated the statistical significance at the level of 0.05 (*p* < 0.05).

To accurately evaluate the color of these millets, color parameters “L” (luminosity), “a” (redness), and “b” (yellowness) values were measured (Figure 2 and Table S1). Significant differences (*p* < 0.05) were detected in the three parameters of the five foxtail millet varieties. NMB had the highest “L” value, meaning the maximum brightness, followed by QZH, YG1, JG21, and DBQ in order. The “a” value of the other four varieties displayed all lower than JG21, especially in NMB, which implied the redness of JG21 was the highest. For the “b” value, the three high eating quality varieties showed deeper yellow than DBQ and NMB. CCI value was obtained by combining the three parameters above, with the highest value in DBQ and the lowest value in NMB. These results were consistent with the visual observation and showed the large discrepancies in millet color among varieties with different eating quality.

### 3.2. Flavonoid Metabolites Profiles in Millets of Different Varieties

**Figure 3:**
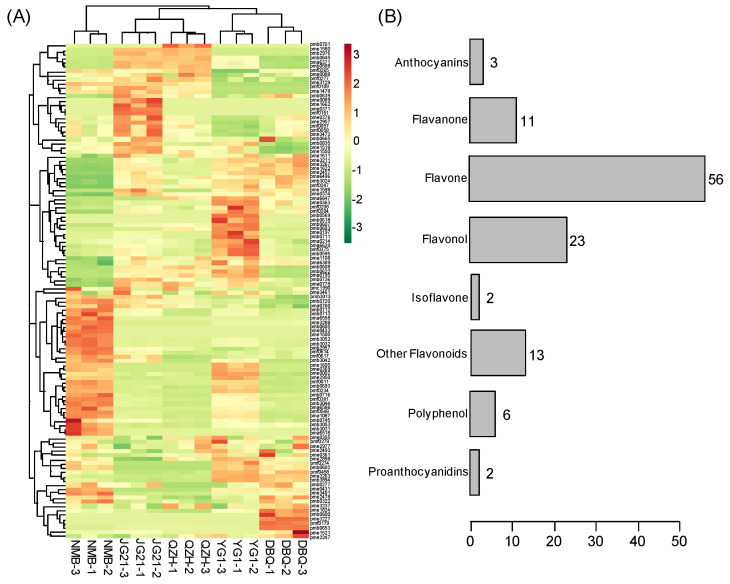
Clustering heat map of all flavonoid metabolites. (**A**) Heatmap of all the detected flavonoid metabolites in the high and poor foxtail millet varieties. (**B**) The numbers and types of flavonoid metabolites in foxtail millet grains.

A total of 116 flavonoid metabolites (Table S2) from the five foxtail millet varieties were identified using UPLC-ESI-MS/MS to obtain the profile of flavonoid metabolites in foxtail millet. The heatmap showed the relative content of flavonoid metabolites after homogenization (Figure 3A). The 116 flavonoid metabolites can be divided into eight groups, including 56 flavones, 23 flavonols, 11 flavanone, 6 polyphenol, 3 anthocyanins, 2 proanthocyanidins, 2 isoflavones, and 13 other flavonoids (Figure 3B). Apigenin, luteolin, naringenin, kaempferol, chrysoeriol, naringenin, and their derivatives made up most of the total flavonoid metabolites, mainly involved four main clusters. The heat map also showed a strong contrast in the relative content of metabolites between NMB and the other four varieties. The relative content of metabolites in JG21 was similar to QZH, and YG1 was similar to DBQ.

**Figure 4:**
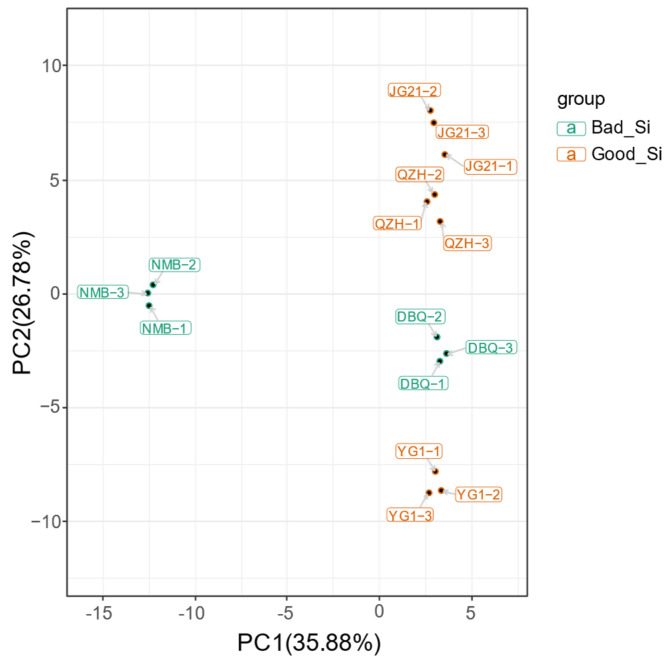
The PCA score plot of flavonoid metabolites in different foxtail millet samples. Good_Si represents high-quality varieties, and Bad_Si represents poor-quality varieties.

We conducted a multivariate statistical analysis of 116 metabolites using PCA. A model with two principal components was constructed. The contribution rates of PC1 and PC2 were 35.88% and 26.78%, respectively. The total contribution rate reached 62.66% (Figure 4). The five varieties were separated clearly, and the replicates were compactly gathered, showing ideal repeatability and reliability. For PC1, four varieties (JG21, QZH, DBQ, and YG1) got similar scores and were significantly separated from NMB, indicating the metabolites profiling of NMB was notably distinguished from the other four varieties. This was consistent with the row-clustering of the heatmap. For PC2, the score of NMB was close to DBQ, and JG21 was close to QZH, caused by their common flavonoids. However, YG1 was located away from the other two high-quality varieties, which might be caused by the large difference in the content of certain flavonoids.

### 3.3. Pairwise Comparison of Flavonoid Metabolites Between High and Poor Eating Quality VarietiesBased on OPLS-DA Model

**Figure 5:**
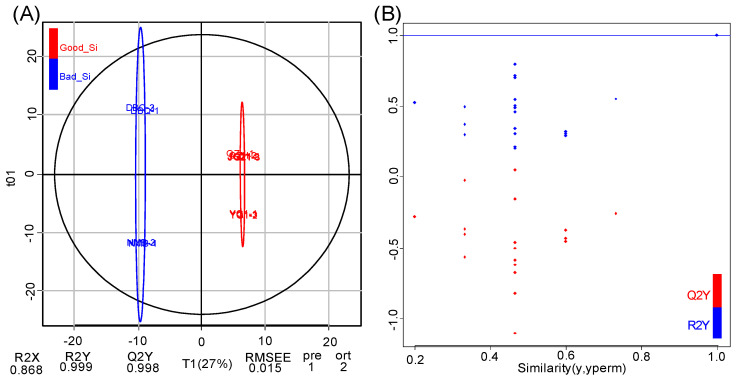
Pairwise comparison based on OPLS-DA model. (**A**) OPLS-DA model plots for the comparison group Good_Si vs. Bad_Si. (**B**) The permutation test of the OPLS-DA model. The x-axis represents the similarity, which is the proportion consistent with the order of the Y variables of the original model. The y-axis represents Q^2^ and R^2^.

To find differential metabolites between high and poor eating quality varieties, we performed the OPLS-DA model to produce pairwise comparisons. In this model, the systematic variation in variable X was decomposed into two parts: linear correlation to Y and orthogonality to Y [35]. It can complete the prediction of sample categories by establishing the relational model between the flavonoid metabolites and sample categories and maximize the distinction between groups mainly reflected in T1 (Figure 5A). In this study, the flavonoid metabolites in high and poor-eating quality millets were explicitly separated based on the OPLS-DA model, showing a significant difference between the two groups. The explanation degree of the principal component to the independent variable X and categorical variable Y was 86.8% (R2X) and 99.9% (R2Y), respectively. The prediction indicator of the model on the five foxtail millet varieties was 99.8% (Q2 = 0.998). The OPLS-DA model was tested by permutation analysis (Figure 5B). As the similarity gradually decreased, the R^2^ and Q^2^ of the random model gradually decreased, indicating no over-fitting phenomenon in the original model. Therefore, these two groups could be used to screen differential flavonoid metabolites.

### 3.4. Identification of Differential Flavonoid Metabolites

**Figure 6:**
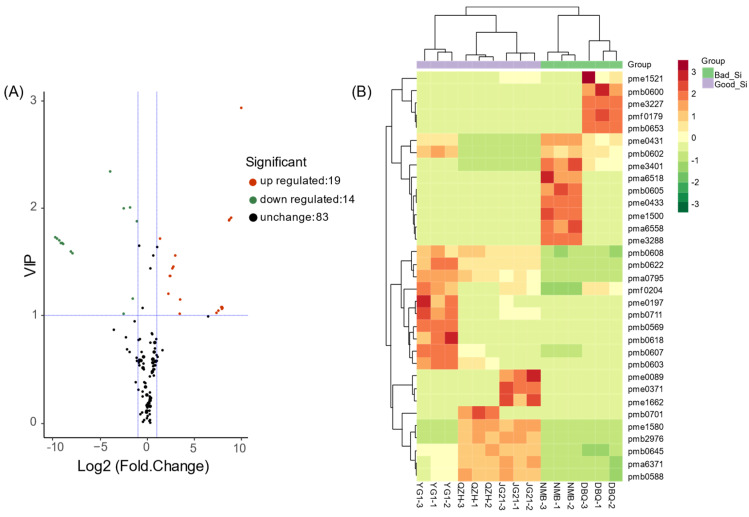
Identification of differential flavonoid metabolites between Good_Si and Bad_Si groups. (**A**) The volcano plot of differential flavonoid metabolites. Each point in the graph represents a metabolite. The red dots represent up-regulated metabolites, the green dots represent down-regulated metabolites, and the black dots represent metabolites with insignificant differences. (**B**) Clustering heat map of differential flavonoid metabolites. Columns represented samples, and metabolites were represented by rows. The data of metabolite content were normalized by the method of maximum difference normalization. The up-regulated and down-regulated metabolites were represented with red and green, respectively.

We screened differential flavonoid metabolites between high and poor eating quality varieties by combining the fold-change (FC) and variable importance in project values (VIP). There were 33 differential flavonoid metabolites (19 up-regulated, 14 down-regulated) including 21 flavones, six flavonols, two flavanones, two proanthocyanidins, and two other flavonoids in high-quality varieties compared with poor quality varieties, with |log2 FC| > 2 and VIP > 2 (Table S3). The volcano plot could broadly display the number of differential flavonoids metabolites and the variation between high and poor eating quality foxtail millet varieties (Figure 6A). These metabolites might represent the differential flavonoid metabolites in foxtail millet varieties that caused different eating qualities.

We conducted HCA to compare the differences in 33 flavonoid metabolites among varieties. These metabolites were presented as a heatmap after normalization (Figure 6B). As shown in the heatmap, specific metabolites in each variety were displayed intuitively. All of the 33 flavonoid metabolites in the different varieties could be divided into two categories. NMB and DBQ were clustered into one category, whereas JG21, QZH, and YG1 were clustered into the other, in which JG21 and QZH shared more similarities. Luteolin and its derivatives (pma6371, pmb0622, pmb0588), chrysoeriol O-malonylhexoside (pmb0608), and tricetin O-malonylhexoside (pma0795) were the metabolites more abundant in three high-quality varieties. Syringetin 5-O-hexoside (pmb0569), 8-C-hexosyl-hesperetin O-hexoside (pmb0618), procyanidin A1 (pme0431), and syringetin 7-O-hexoside (pmb0602) were the main differential flavonoid metabolites between YG1 and the other two high-quality varieties. Rutin (pme0197), showing the highest content in YG1 and lower in the other four varieties, was a derivative of quercetin and pertained to flavonol. Proanthocyanidins (pme0431, pme0433) were mainly detected in NMB and DBQ.

### 3.5. Enrichment Analysis Clarified the Metabolic Pathways of Differential Flavonoid Metabolites

**Figure 7:**
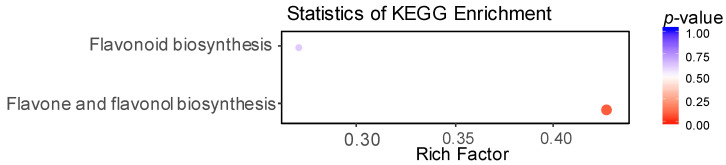
The KEGG classification of differential flavonoid metabolites. The x-axis indicates the Rich Factor, and the y-axis indicates the names of metabolic pathways. The color of the dot means *p*-value, the darker red, the greater degree of KEGG enrichment, and the size of the dot means the numbers of the differential metabolites.

The differential flavonoid metabolites were mapped to the Kyoto Encyclopedia of Genes and Genomes (KEGG) database (http://www.genome.jp/kegg/, accessed on 2019). We mainly identified two metabolic pathways, the flavonoid biosynthesis pathway (ko00941) and one of its branches, flavone and flavonol biosynthesis pathway (ko00944), at a 0.05 level (*p*-value) (Figure 7, Table S3). The specific flavonoid metabolites in high eating quality varieties were mainly distributed in the flavonoid biosynthesis pathway (ko00941). In contrast, specific flavonoid metabolites in poor eating quality varieties were distributed primarily on the flavone and flavonol biosynthesis pathway (ko00944).

**Figure 8:**
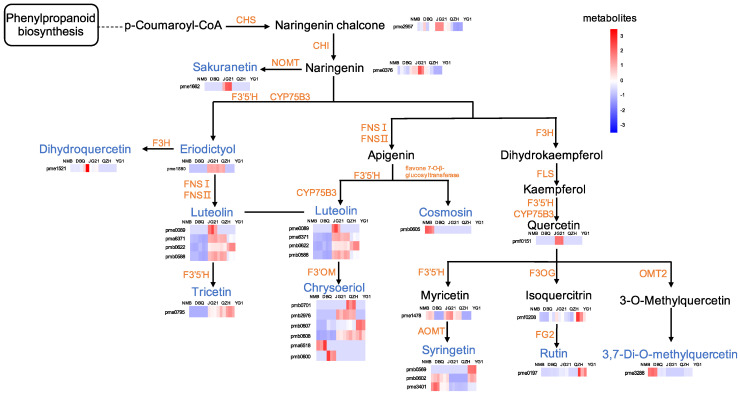
The pathway of differential flavonoid metabolites. Blue letters represent differential metabolites. Orange letters represent enzymes in metabolic pathways. A rectangle with a particular color represented the relative content of each metabolite. As shown in the upper-right bar, the up-regulated and down-regulated metabolites were represented with red and blue, respectively. CHS: chalcone synthase C2; CHI: chalcone isomerase; F3′5′H: flavanoid 3′,5′-hydroxylase; NOMT: naringenin 7-O-methyltransferase; CYP75B3: flavonoid 3′-monooxygenase; F3H: flavanone 3-dioxygenase; FNSI: flavone synthase I; FNSII: flavone synthase II; FLS: flavonol synthase; FOM: flavone 3′-O-methyltransferase; AOMT: flavonoid 3′,5′-methyltransferase; F3OG: flavonol 3-O-glucosyltransferase; FG2: flavonol-3-O-glucoside L-rhamnosyltransferase; OMT2: quercetin 3-O-methyltransferase.

Based on the KEGG database, we constructed a foxtail millet flavonoid metabolic network containing 33 differential flavonoid metabolites, with approximately two branches from naringenin (Figure 8). In this network, most enzymes might possess functions in metabolite modification when most of the detected flavonoids were decorated forms by glycosyltransferase, hydroxylase, and methyltransferase. According to the network, flavonoid 3′,5′-hydroxylase (F3′5′H), flavone synthase I (FNSI), flavone synthase II (FNSII), flavonoid 3′-monooxygenase (CYP75B3), flavone 3′-O-methyltransferase (F3′OM), flavonoid 3′,5′-methyltransferase (AOMT), flavone 7-O-β-glucosyltransferase, flavonol 3-O-glucosyltransferase (F3OG), flavonol-3-O-glucoside L-rhamnosyltransferase (FG2) and quercetin 3-O-methyltransferase (OMT2) were the key enzymes in the process of the differential metabolites biosynthesis.

## 4. Discussion

With high nutritional value, foxtail millet has received more and more attention from consumers. According to the “Compendium of Materia Medica” (a book on traditional Chinese medicine, written in 1578), the millet porridge is exceedingly nourishing for the stomach and effective for children’s diarrhea. The yellow color is the primary indicator for consumers to choose millet, and carotenoids and flavonoids are the dominating yellow pigments in dehulled foxtail millet. Studies have shown that the accumulation of carotenoids and flavonoids contributes to the seeds’ palatability and nutritional value [36,37]. As an important provitamin A, carotenoids can be metabolized to retinol, effectively preventing vision loss [38]. By comparing the transcript levels of carotenoid structural genes between green and yellow color millets, the role of carotenoids in the formation of yellow millet color has been proven [39]. Flavonoids also affect the color, nutritional value, and antioxidant properties of plants and possess a wide range of healthcare activities and pharmacological functions [40]. The studies on flavonoids in plants have also provided a theoretical basis for its application in medicine, healthcare, food, and cosmetics [41]. Therefore, it is of great importance to explore the flavonoids in foxtail millet.

Studies have shown the content of flavonoids varied among different species and different varieties of the same species [42,43]. It was reported that wheat varieties with different flavonoid content resulted in the difference in bread taste. Apigenin was only identified in the poor wheat variety [44], which was consistent with our findings. This could be applied to wheat breeding and improve wheat varieties using molecular breeding approaches. The flavonoid metabolites in different buckwheat varieties also provided a theoretical basis for the sufficient utilization of special buckwheat varieties [45]. Flavonoids in plant leaves play vital roles in defending against abiotic stresses [46]. Researchers have clarified the accumulation and variations of more than 300 metabolites in the leaves of foxtail millet at three-leaf and five-leaf stages [47]. Still, there is little research on the flavonoids in millet grains. In our study, flavonoid metabolites in foxtail millet varieties of different qualities were investigated. The three high eating quality varieties, JG21, QZH, and YG1 contained similar flavonoid contents. They were significantly different from NMB and DBQ, which provided a theoretical basis for the utilization of functional ingredients in foxtail millet.

According to the comparison group, luteolin, quercetin, and their derivatives were mainly present in three high-eating quality varieties. As a flavone, luteolin is one of the most common flavonoids in edible plants, and it has been found in different fruits and vegetables, including carrots, peppers, and celery [48]. Recent research pointed out that luteolin might be a promising molecule for developing topic formulations and systemic agents against inflammatory skin diseases. It has also been suggested as a cancer chemopreventive agent [49,50]. In our study, luteolin was abundant in three high-quality foxtail millet varieties but absent in the other two varieties. Quercetin, belonging to flavonols, has been asserted to have many valuable effects on health, including preventing diseases such as lung cancer, osteoporosis, and inflammatory disorders [51,52]. It is a dietary flavonoid found in fruits (mainly citrus), many seeds (buckwheat), and so on [53]. Present studies have provided evidence that quercetin improves gut health and helps alleviate metabolic disorders, which is likely to be one reason why the millet porridge is effective for children’s diarrhea [54]. Most of the quercetin existed in YG1, and a small amount was detected in JG21. This study showed that the seven glycoside derivatives of chrysoeriol were mainly accumulated in JG21, QZH, YG1. The metabolites mentioned above might be part of the high eating quality of JG21, QZH, and YG1.

Although exhibiting poor eating quality, specific flavonoids with pharmacological functions were also detected in NMB and DBQ. Procyanidins existed mainly in NMB, with YG1 and DBQ containing just a minute quantity of procyanidin A_1_. Previous studies have shown that procyanidins exert physiological and cellular activities, facilitate homeostasis, and possess anti-inflammatory effects in vitro and in vivo [55]. However, procyanidins gave the grape wine astringent taste [56,57], and this might also be a reason for the astringent flavor of NMB. Apigenin 7-O-glucoside (pmb0605) and di-C, C-hexosyl-apigenin (pmb0605) were glycoside compounds of apigenin, whereas the former mainly accumulated in NMB and the other mainly in DBQ. As a dietary flavonoid, apigenin is widely distributed in plants like celery, parsley, and chamomile, which has shown an interesting link between diet and treating chronic diseases, including cancer [58,59]. Foxtail millets with poor quality may also have a specific health role. Therefore, DBQ and NMB represent valuable resources for the improvement of foxtail millets.

The flavonoid metabolic network we proposed here was based on the differential flavonoid metabolites. We specified the key enzymes that might regulate the biosynthesis of the differential flavonoid metabolites. However, the genes encoding these enzymes and their regulation mechanism of the whole flavonoid metabolic network need to be further studied. *CitF3H* in citrus plants was verified to convert naringenin into dihydrokaempferol, which contributed to the genetic improvement of citrus plants and the synthesis of beneficial flavonoids [43]. The functions of flavonoid-related enzyme genes in foxtail millet need to be verified. An enzyme (TraesCS1A01G347100) in wheat kernels has been shown to function on flavonoids (apigenin, kaempferol, naringenin) to produce various glycosylation products by in vitro enzymatic validation [60]. Derivatives of metabolites from different varieties also differed. For instance, the contents of four derivatives of chrysoeriol (pmb0701, pmb2976, pmb0607, pmb0608) were higher in high eating quality varieties, whereas pma6518 and pmb0600 were mainly found in two poor eating quality varieties, the enzymes that play catalytic roles in which need to be identified. In this flavonoid metabolic network, one gene may be involved in regulating the biosynthesis of multiple metabolites, or on the contrary, multiple genes may also be related to the same flavonoid. At present, a rapid-cycling mini foxtail millet mutant, *xiaomi*, has provided us with a good resource for deeper exploration of these genes [61]. Moreover, the complex structure of flavonoids poses a considerable challenge to their identification. Therefore, precise structure determination of the metabolites will help enlighten a complete network.

Flavonoid metabolites varied greatly in the tested foxtail millet varieties, and each of them accumulated its unique metabolites, which might provide these varieties with potentially medical functions. Additionally, the result of this study identified the main biosynthesis pathways involved in these differential metabolites. Combing the whole regulation networks of flavonoids metabolism could provide a theoretical basis for researching the quality and the breeding for high-quality varieties in foxtail millet.

## 5. Conclusions

In summary, we initially identified 116 flavonoid compounds in foxtail millet. Then we identified 33 differential flavonoid metabolites between high and poor eating quality varieties. By further analyzing these 33 metabolites, we found specific metabolites in each variety, such as the luteolin in three high eating quality varieties, the quercetin in YG1, and the procyanidins in NMB. Finally, we combined the differential metabolites with the KEGG database and found the key enzymes that might regulate the biosynthesis of these metabolites. This project provides information for the study of flavonoid biosynthesis in foxtail millet. It will also lay a theoretical foundation for the breeding of high-quality foxtail millet varieties.

## Data Availability

Not applicable.

## Supplementary Materials

The following are available online at https://www.mdpi.com/article/10.3390/life11060578/s1, Table S1: Color parameter values of different foxtail millet varieties, Table S2: A list of the 116 flavonoid metabolites detected in the five foxtail millet varieties, Table S3: A list of the 33 differential flavonoid metabolites between high and poor eating quality varieties.
